# Investigation of Marine-Derived Natural Products as Raf Kinase Inhibitory Protein (RKIP)-Binding Ligands

**DOI:** 10.3390/md19100581

**Published:** 2021-10-18

**Authors:** Shraddha Parate, Vikas Kumar, Jong Chan Hong, Keun Woo Lee

**Affiliations:** 1 Division of Applied Life Science, Plant Molecular Biology and Biotechnology Research Center (PMBBRC), Gyeongsang National University (GNU), 501 Jinju-daero, Jinju 52828, Korea; parateshraddha@gmail.com; 2 Division of Life Sciences, Department of Bio & Medical Big Data (BK4 Program), Research Institute of Natural Sciences (RINS), Gyeongsang National University (GNU), 501 Jinju-daero, Jinju 52828, Korea; vikaspathania777@gmail.com

**Keywords:** RKIP, marine natural products, pharmacophore modeling, virtual screening, molecular docking, molecular dynamics simulations, binding free energy

## Abstract

Raf kinase inhibitory protein (RKIP) is an essential regulator of the Ras/Raf-1/MEK/ERK signaling cascade and functions by directly interacting with the Raf-1 kinase. The abnormal expression of RKIP is linked with numerous diseases including cancers, Alzheimer’s and diabetic nephropathy. Interestingly, RKIP also plays an indispensable role as a tumor suppressor, thus making it an attractive therapeutic target. To date, only a few small molecules have been reported to modulate the activity of RKIP, and there is a need to explore additional scaffolds. In order to achieve this objective, a pharmacophore model was generated that explores the features of locostatin, the most potent RKIP modulator. Correspondingly, the developed model was subjected to screening, and the mapped compounds from Marine Natural Products (MNP) library were retrieved. The mapped MNPs after ensuing drug-likeness filtration were escalated for molecular docking, where locostatin was regarded as a reference. The MNPs exhibiting higher docking scores than locostatin were considered for molecular dynamics simulations, and their binding affinity towards RKIP was computed via MM/PBSA. A total of five molecules revealed significantly better binding free energy scores than compared to locostatin and, therefore, were reckoned as hits. The hits from the present in silico investigation could act as potent RKIP modulators and disrupt interactions of RKIP with its binding proteins. Furthermore, the identification of potent modulators from marine natural habitat can act as a future drug-discovery source.

## 1. Introduction

Raf kinase inhibitory protein (RKIP), also recognized as phosphatidylethanolamine-binding protein 1 (PEBP1), is an evolutionarily conserved, small (23 kDa) cytosolic protein, originally purified from bovine brain [1]. RKIP is broadly expressed in normal human tissues and identified to have an essential role in numerous physiological processes including neural development, spermatogenesis, cardiac output and membrane biosynthesis [2]. RKIP has been shown to be a vital modulator of various cell signaling cascades including the G protein-coupled receptor (GPCR), mitogen-activated protein kinase (MAPK) and the nuclear factor κB (NF-κB) pathways [1,2]. In particular, RKIP was acknowledged as an endogenous regulator of the kinases involved in the aforementioned pathways. RKIP binds specifically to the cytoplasmic serine/threonine Raf-1 kinase [3] and obstructs the Raf-1 dependent activation of MAPK/extracellular signal-regulated kinase (ERK) kinase (MEK), thereby disturbing the activation of ERK [4]. Additionally, RKIP indirectly hampers GPCR, which is an upstream activator of Raf-1. Therefore, when RKIP is released from Raf-1 after phosphorylation by protein kinase C (PKC) at the Ser153 residue, it associates with the kinase involved in the GPCR pathway, G protein-coupled receptor kinase 2 (GRK2) [5]. The phosphorylated RKIP/GRK2 association results in an enhanced activation of GPCR, thereby contributing to the overactivation of MAPK and downstream targets, as Raf-1 will no longer be inhibited by RKIP. Moreover, RKIP can act as a negative modulator of NF-κB signaling pathway by associating with upstream kinases NIK (NF-κB inducing kinase), TAK (transforming growth factor beta (TGFB)-activated kinase 1), IKKα (inhibitory-κ kinase α), IKKβ (inhibitory-κ kinase β) and inhibiting their kinase activity [4].

Owing to its essential role as an intracellular signaling pathway modulator, the dysregulated RKIP expression is implicated in several diseases, including cancer [6,7]. Literature reviews suggested the association of RKIP with prostate cancer [8], glioma [9], breast cancer [10], melanoma [11], colorectal cancer [12], lung cancer [13], thyroid cancer [14] and nasopharyngeal carcinoma [15]. Additionally, dysregulated PEBP1 expression was also observed to contribute to Alzheimer’s disease (AD) [16] and diabetic nephropathy [1,2]. Interestingly, RKIP was also identified as being a metastasis suppressor [17]. Subsequently, RKIP has become a novel diagnostic marker for the associated pathologies. It is, therefore, imperative to search for RKIP agonists or inhibitors, which might aid in developing drugs to treat cell signaling-related abnormalities. The development of new probes for RKIP will help in the effort of perturbing RKIP’s function and to define its seemingly conflicting roles. 

**Figure 1:**
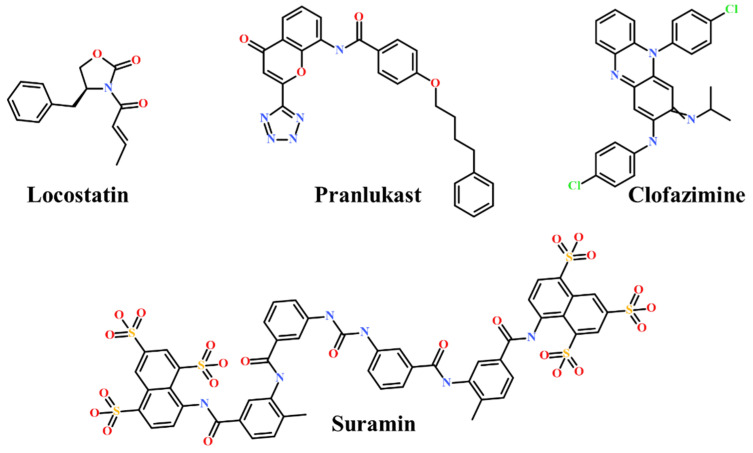
Chemical structures of small molecule RKIP modulators identified to date.

Presently, only a few small molecules have been identified to modulate RKIP’s role in pathological illnesses by binding to its conserved ligand-binding pocket. This pharmacological modulation has been accomplished through drugs encompassing Locostatin [18,19], pranlukast [20], clofazimine [21] and suramin [22] (Figure 1). The non-antibacterial oxazolidinone derivative, UIC-1005, was identified as a cell sheet migration inhibitor of RKIP [23] and later renamed as locostatin after its capability to inhibit cell locomotion in multiple systems [24]. In particular, locostatin abrogates the ability of RKIP to interact with Raf-1 kinase and also with GRK2, thereby functioning as a protein–protein interaction inhibitor [18,19]. Additionally, Sun et al. reported a novel RKIP-binding ligand, pranlukast, via structure-based virtual screening and demonstrated its binding on the conserved ligand-binding RKIP pocket through NMR and fluorescence experiments [20]. Guo et al. and team additionally identified Clofazimine and Suramin binding to RKIP through a combination of NMR and molecular docking [21,22].

As secondary metabolites of microbes, plants, animals and marine organisms, natural products play predominant roles in self-defense, physiological homeostasis and propagation [25]. Moreover, they are prolific sources of active constituents in therapeutic drugs, featuring more structural diversity and complexity, fewer nitrogen or halogen atoms, more stereogenic centers and greater druggable pharmacophores than compared to synthetic molecules [26]. Marine organisms can be considered as the most abundant source of bioactive natural products as the diverse structures obtained from them reflect biodiversity of genes, species and ecosystems [27]. Drug discovery from marine natural products (MNP) has seen a resurgence in the past years with a growing number of molecules entering clinical trials [28,29]. A recent literature survey revealed strong anticancer biological activities concerning 170 MNPs and their semi-synthetic analogues [30]. MNPs have also exhibited neuroprotective effects on therapeutic targets of AD, Parkinson’s disease (PD) and ischemic brain stroke [31].

The small number of RKIP-binding ligands in the literature and the structural diversity of compounds acquired from marine natural habitat prompted us to further explore potential therapeutics targeted for RKIP-related ailments. Accordingly, in the present in silico study, RKIP-binding ligands were identified via auto pharmacophore-based virtual screening of MNPs. Correspondingly, a pharmacophore model was generated by exploiting the features of a small molecule RKIP inhibitor, locostatin. Since locostatin has demonstrated exceptional results as an RKIP inhibitor, we intended to exploit the pharmacophore features manifested by its chemical scaffold. Subsequently, the attained model was escalated to screen the MNP library. The pharmacophore-mapped drug-like MNPs were further docked with the molecular structure of RKIP, and the compounds demonstrating better docking scores than locostatin were refined by computational simulations under physiological conditions. The MNPs exhibiting significantly better binding affinity scores than locostatin, as computed by Molecular Mechanics Poisson–Boltzmann Surface Area (MM/PBSA), were confirmed as hits and reported as potential therapeutics for RKIP-related diseases.

## 2. Results

**Figure 2:**
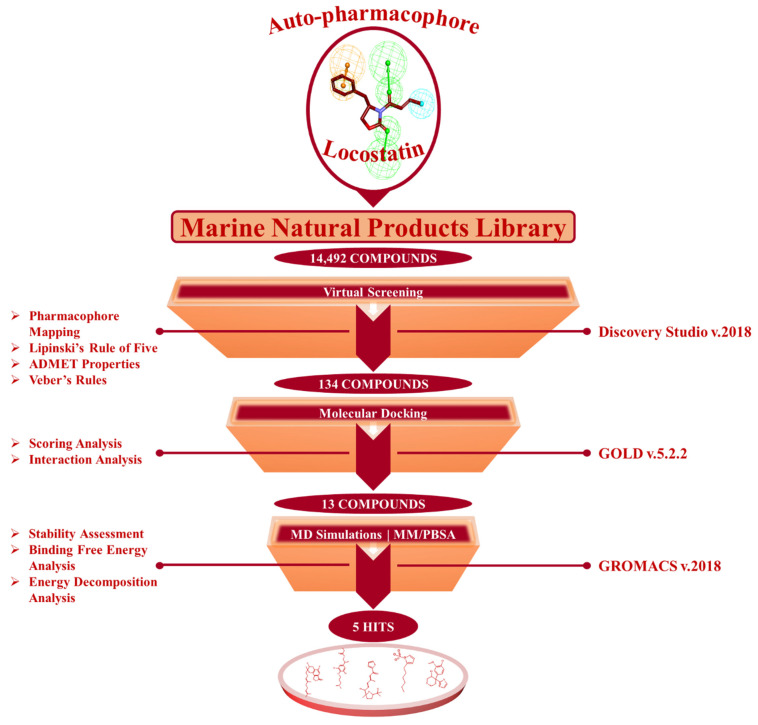
The in silico workflow depicting the sequence of computational techniques for identification of RKIP modulators.

The present investigation applied a sequence of computational methods for the identification of RKIP modulators via pharmacophore modeling from a single ligand structure of locostatin by using the below summarized workflow (Figure 2). 

### 2.1. Generated Auto-Pharmacophore Model

**Figure 3:**
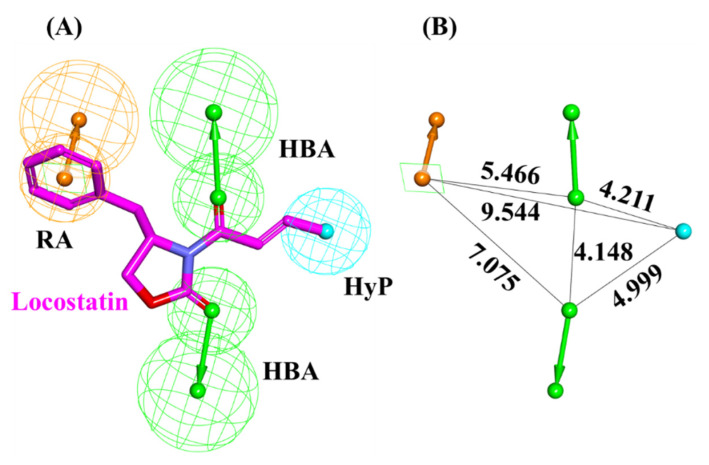
Auto-pharmacophore model exploiting locostatin. (**A**) Pharmacophore features demonstrated by locostatin- HBA (hydrogen bond acceptor), HyP (hydrophobic) and RA (ring aromatic). (**B**) Interfeature distance between the mapped features of locostatin.

A pharmacophore model was generated utilizing locostatin, the most potent RKIP inhibitor [18,19]. Prior to model generation, the *Feature Mapping* protocol in DS identified eight features encompassing four hydrogen bond acceptor (HBA), two hydrophobic (HyP) and two ring aromatic (RA) as the most occurring ones in locostatin. Subsequently, the generated model revealed a total of four features, with 2HBA, 1HyP and 1RA representing the most indispensable features of locostatin (Table 1). Upon scrupulous examination of the superimposed model on locostatin, it was observed that the 2-oxazolidinone core complements both the HBA features, the crotonyl moiety complements the HyP feature and the benzyl moiety complemented the RA feature [32] (Figure 3).

### 2.2. Drug-Like Marine-Derived Compounds from Virtual Screening

From a total of 14,492 compounds available in the MNP library, the auto-pharmacophore model generated from the above analysis mapped an aggregate of 2557 MNPs representing the same features as acquired from locostatin. The large number of mapped compounds was further reduced by subsequent filtration on the basis of Lipinski’s Rule of Five (Ro5) and Veber’s rule. A total of 889 MNPs followed the collective Ro5 and Veber’s rules demonstrating molecular weight <500 kDa, number of hydrogen bond donors ≤5, number of HBA ≤10, compound’s lipophilicity (logP) ≤5 and number of rotatable bonds ≤10 [33,34]. Additionally, the evaluation of ADMET (absorption, distribution, metabolism, excretion and toxicity) properties further reduced the total number of compounds to 134 drug-like MNPs (Figure 2). These 134 MNPs displayed no blood–brain barrier (BBB) permeability, no CYP2D6 binding, no hepatotoxicity, good intestinal absorption and aqueous solubility. The procured 134 drug-like MNPs were escalated for molecular docking with the RKIP ligand-binding pocket.

### 2.3. Molecular Docking of Screening-Derived Compounds with RKIP

Molecular docking studies divulge into crucial information regarding the binding mode of ligands in the target protein pocket, thereby elucidating on the protein–ligand interaction. The validation of docking parameters resulted in reproducing similar docked poses as that observed for the co-crystallized PTR (Figure S1), establishing the efficiency of GOLD. The virtually screened 134 marine compounds were docked with RKIP along with locostatin as the reference (REF) compound. The REF compound demonstrated a Goldscore of 48.64 and a Chemscore of −26.48, while a total of thirteen drug-like MNPs exhibited higher Goldscores and lower Chemscores than compared to REF (Table 2). The thirteen compounds also displayed interactions with the key residues of RKIP ligand-binding pocket encompassing Asp70, Ala73, Pro74, Tyr81, Trp84, His86, Gly108, Gly110, Pro112, His118, Tyr120, Tyr181 and Leu184. Therefore, the stability of these compounds and the REF was confirmed in the RKIP ligand-binding pocket via processing them for molecular dynamics (MD) simulations.

### 2.4. Molecular Dynamics Simulation Analyses

**Figure 4:**
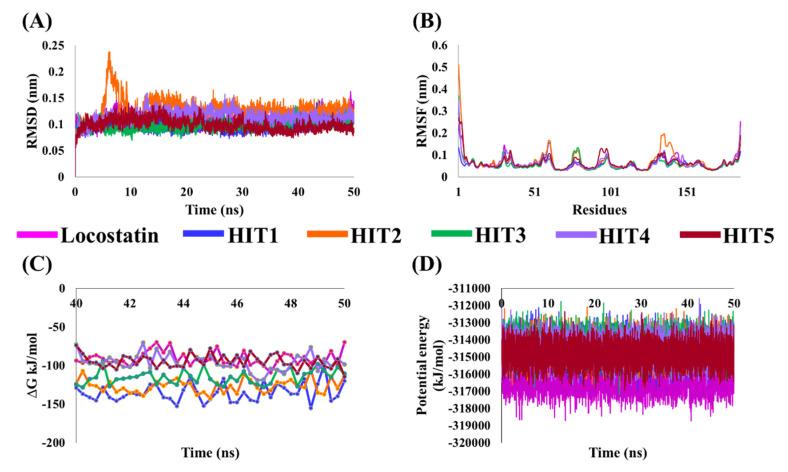
Molecular dynamics (MD) simulation analyses plots demonstrating the (**A**) backbone root mean square deviation (RMSD), (**B**) backbone root mean square fluctuation (RMSF), (**C**) binding free energy (∆G_bind_) values and (**D**) potential energy of the reference (REF) compound, locostatin and identified hits with RKIP.

MD simulations were executed for the thirteen identified MNPs and REF, docked with RKIP, to elucidate their dynamic behavior at the physiological level. Along with performing MD simulations, the binding free energies (BFE) were also computed to assess the binding affinity of each ligand towards RKIP. This was instigated by the ‘*g_mmpbsa*’ program, and the BFE scores of thirteen compounds were computed (Table S1). The REF compound, locostatin, exhibited a BFE score of −90.909 ± 9.155 kJ/mol, while five MNPs revealed better BFE scores. Therefore, the five MNPs were regarded as hits and were ranked according to their BFE scores (Table 3, Figure 4C).

**Figure 5:**
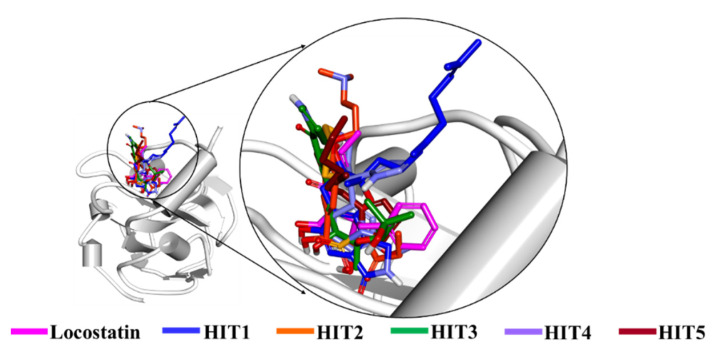
Binding mode of reference (REF) compound, locostatin and identified hits in the ligand-binding pocket of RKIP.

The stability of hits and REF was determined on the basis of their backbone root mean square deviation (RMSD), root mean square fluctuation (RMSF) and potential energy plots. As perceived from the RMSD plots, it was observed that all the systems remained stable throughout the period of 50 ns, except for HIT2 which displayed slight instability towards the 6 ns (Figure 4A). The RMSF analysis also demonstrated the stability of all residues for the entire 50 ns of simulation run with an exception of HIT2, for which its residues (Asp134-Ser142) exhibited minor fluctuation (Figure 4B). Additionally, the energy of all the six systems remained stable as perceived from their potential energy plots (Figure 4D). In order to further gain insight into their mode of binding at the ligand-binding pocket of RKIP, the representative structures were extracted from the last 10 ns of stable MD trajectories and superimposed. The hits exhibited a similar binding mode as that observed for the locostatin (Figure 5).

**Figure 6:**
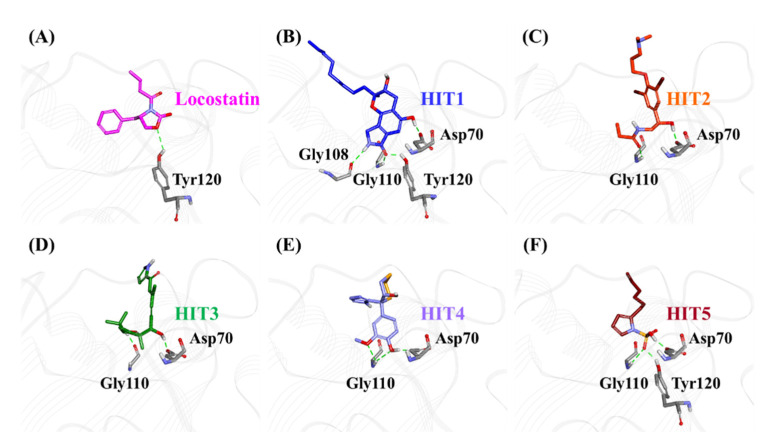
The three-dimensional (3D) intermolecular interactions of (**A**) reference (REF) compound, locostatin and the (**B**−**F**) identified hits with the key residues of RKIP. The hydrogen bonding interactions are displayed as dashed green lines.

The characteristic binding interaction of the five hits and locostatin was examined on the basis of the average structure extracted from last 10 ns. The REF compound, locostatin, was observed to demonstrate one hydrogen bond with residue Tyr120 (bond length: 2.73 Å) (Figure 6A). In addition, REF also formed hydrophobic bonds with residues Trp84 (π–π stacked, bond length: 4.48 Å; π-alkyl, bond length: 4.97 Å), Val107 (π-alkyl, bond length: 5.05 Å) and Tyr181 (π–π T-shaped, bond length: 4.93 Å) (Figure S2A). The residues Asp70, Ala73, Pro74, Tyr81, His86, Gly108, Gly110, Pro111, Pro112, His118, Leu180 and Leu184 also supported locostatin, characterized by carbon–hydrogen bonds and van der Waals interactions (Figure S2A). 

The representative structure of HIT1 demonstrated hydrogen bonds with four RKIP residues: Asp70 (bond length: 1.70 Å), Gly108 (bond length: 2.28 Å), Gly110 (bond length: 1.86 Å) and Tyr120 (bond length: 1.80 Å) (Figure 6B). Additionally, HIT1 formed hydrophobic bonds with residues Trp84 (π–π stacked, bond length: 4.75 Å), Gly110 (amide π-stacked, bond length: 4.89 Å), Pro112 (alkyl, bond length: 4.51 Å), Tyr181 (π-alkyl, bond length: 4.10 Å) and Leu184 (alkyl, bond length: 5.13 Å) (Figure S2B). The residues Asp72, Ala73, Pro74, Tyr81, His86, Val107, Ser109, Pro111, His118, Gly143, Leu180 and Ser185 also assisted in the binding of HIT1 with RKIP via carbon–hydrogen bonds and van der Waals interactions (Figure S2B).

The average structure acquired for HIT2 exhibited hydrogen bonds with two RKIP residues: Asp70 (bond length: 1.68 Å) and Gly110 (bond length: 2.09 Å) (Figure 6C). Moreover, HIT2 exhibited hydrophobic interactions with Ala 73 (alkyl, bond length: 4.31 Å; π-alkyl, bond length: 5.17 Å), Pro74 (alkyl, bond length: 4.08 Å), Tyr81 (π-sigma, bond length: 3.74 Å), Trp84 (π-alkyl, bond length: 4.71 Å; π-alkyl, bond length: 5.28 Å; π-alkyl, bond length: 4.17 Å), Pro112 (π-alkyl, bond length: 5.09 Å) and Tyr181 (π-alkyl, bond length: 4.62 Å; π-alkyl, bond length: 5.40 Å) residues (Figure S2C). The carbon–hydrogen bonds, π-donor hydrogen bonds and van der Waals interactions with residues Pro74, Trp84, His86, Val107, Gly108, Ser109, Pro111, His118 and Tyr120 also played a vital role in supporting HIT2 in RKIP binding pocket (Figure S2C).

The representative HIT3 structure displayed hydrogen bonds with Asp70 (bond length: 1.69 Å) and Gly110 (bond length: 1.69 Å) (Figure 6D). The residues Pro74 (alkyl, bond length: 4.28 Å), Tyr81 (π-alkyl, bond length: 5.23 Å), Trp84 (π-alkyl, bond lengths: 3.53 Å and 4.46 Å), His86 (π-alkyl, bond lengths: 4.50 Å and 4.96 Å) and Tyr120 (π-alkyl, bond length: 5.38 Å) established hydrophobic bonds with RKIP (Figure S2D). Furthermore, residues Ala73, Val107, Ser109, Pro111, Pro112, His118, Val177, Leu180, Tyr181 and Leu184 also held a crucial role in assisting HIT3 via van der Waals interactions (Figure S2D).

The structure for HIT4, extracted as representative, exhibited hydrogen bonds with Asp70 (bond length: 1.68 Å) and Gly110 (bond lengths: 2.46 Å and 2.57 Å), similar to HIT2 and HIT3 (Figure 6E). Numerous types of hydrophobic bonds were formed by residues- Ala73 (π-alkyl, bond length: 4.98 Å), Tyr81 (π-alkyl, bond length: 4.95 Å), Trp84 (π-π stacked, bond length: 4.90 Å), Gly110 (amide-π stacked, bond length: 4.05 Å), Pro112 (alkyl, bond length: 5.49 Å) and Tyr181 (π–π T-shaped, bond length: 4.66 Å) (Figure S2E). The residue His86 established a carbon–hydrogen bond, while Val107, Gly108, Ser109, Pro111, His118, Tyr120, Leu180 and Leu184 interacted via van der Waals bonds (Figure S2E).

The representative structure of HIT5 attained from MD analysis showed hydrogen bonds with residues Asp70 (bond length: 1.62 Å), Gly110 (bond length: 2.42 Å) and Tyr120 (bond length: 1.96 Å) (Figure 6F). The residues Trp84 (π–π stacked, bond length: 4.77 Å) and His86 (π-sulfur, bond length: 5.22 Å) established hydrophobic bonds (Figure S2F). Additional residues including His86, Pro112 and His118 supported HIT5 via carbon–hydrogen bonds, while numerous residues such as Ala73, Pro74, Tyr81, Pro111, Tyr181 and Leu184 formed van der Waals interactions (Figure S2F).

The above overall analyses suggests that our hits displayed stability throughout 50 ns of MD run and also formed interactions with vital residues of the RKIP ligand-binding pocket. Most importantly, our hits demonstrated better binding affinity towards RKIP, as observed from their binding free energies. We, therefore, anticipate that our identified hits can provide potential scaffolds as RKIP agonists or inhibitors.

## 3. Discussion

**Figure 7:**
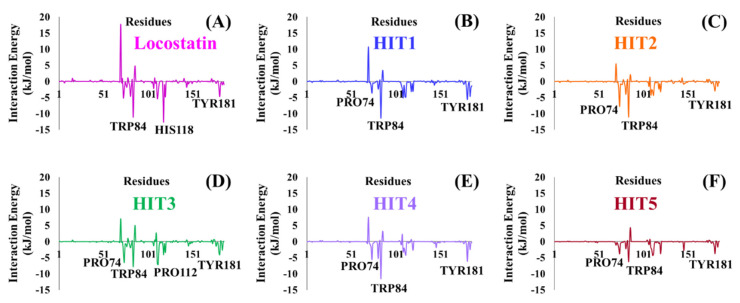
Energy decomposition of individual residues in RKIP contributing to the total binding free energy of (**A**) Locostatin, (**B**) HIT1, (**C**) HIT2, (**D**) HIT3, (**E**) HIT4 and (**F**) HIT5, computed by MM/PBSA.

RKIP/PEBP1 is involved in regulating several signaling pathways including Raf-1/MEK/ERK, NF-κB and GPCR by directly interacting with and inhibiting Raf-1, MEK and ERK protein kinases of the pathways, respectively [35]. RKIP was identified to contribute to dysregulated expression in numerous diseases as well as recognized as being a metastasis suppressor [17]. Only a few RKIP modulators have been identified to date encompassing locostatin, pranlukast, clofazimine and suramin (Figure 1), and there is still a need to search for additional ligands modulating the function of RKIP. Taking these views into account, we pursued our research towards exploring the features of the most potent cell sheet migration inhibitor of RKIP, locostatin [19]. As the X-ray crystallographic structure of RKIP/locostatin is difficult to obtain owing to locostatin’s function to partially aggregate in vitro [18,19], the single structure of locostatin was adapted for our study. Therefore, an auto-pharmacophore model of locostatin was generated, which resulted in a four-featured model (Figure 3). Since marine extracts have displayed a remarkable potential as being a source of new drugs and is a relatively unexplored habitat, a MNP library of 14,492 compounds by Prof. Encinar (http://docking.umh.es/chemlib/mnplib accessed on 21 June 2021) was utilized for our study. Consequently, the library was screened using the pharmacophore model, retrieving a total of 2557 compounds that mapped the features of the pharmacophore. A drug-like database was generated from the above large number of compounds by employing Lipinski’s Ro5, ADMET and Veber’s rules, reducing the number to 134 compounds. The 134 drug-like compounds were taken forward for molecular docking with the crystal structure of RKIP in complex with o-phosphotyrosine (PTR) (PDB ID: 2QYQ) [36]. The RKIP/PTR is the first molecular structure providing a model of how a ligand would possibly bind in the ligand-binding pocket of RKIP [18]. Molecular docking of aforementioned 134 drug-like ligands into the binding pocket of RKIP resulted in the identification of thirteen compounds, which demonstrated better docking scores (Goldscore and Chemscore) than locostatin (Table 2). Moreover, the thirteen compounds also displayed similar interactions with RKIP, as observed for locostatin. Although molecular docking is computationally proficient, its prediction of the protein-ligand binding pose is not usually accurate. Therefore, these compounds were escalated to check their stability in the RKIP binding pocket via MD simulations. The simulations were also supplemented by calculating the binding affinity of each compound towards RKIP, and this was performed via MM/PBSA. The MM/PBSA method has been extensively used to gauge the poses from docking, determine their stability, predict the affinity towards the target protein and also to identify the hotspots responsible for the affinity [37]. From a total of thirteen drug-like compounds, five exhibited better binding affinities towards RKIP than that of locostatin (Table S1). With the locostatin BFE value of −90.909 kJ/mol, HIT1, HIT2, HIT3, HIT4 and HIT5 demonstrated the values of −135.283 kJ/mol, −126.597 kJ/mol, −115.088 kJ/mol, −95.450 kJ/mol and −94.582 kJ/mol, respectively (Table 3). The total number of free energy scores for each RKIP/HIT complex was characterized by individual scores of van der Waals, electrostatic, polar solvation and SASA energy. It was observed that the van der Waals and electrostatic forces played a major role in total binding free energy, thereby explaining that the van der Waals and hydrophobic interactions have a vital role in assisting the binding of hits with RKIP. From the RMSD, RMSF and potential energy analysis, it was perceived that our hits also remained stable in the binding pocket of RKIP (Figure 4). The representative structure was extracted from the last 10 ns of stable trajectory for all hits as well as locostatin, and the interaction pattern was scrutinized. Literature survey revealed that the conserved ligand-binding pocket of RKIP can be defined by 16 residues at the protein surface: Asp70, Ala73, Pro74, Tyr81, Trp84, His86, Val107, Gly108, Gly110, Pro111, Pro112, His118, Tyr120, Leu180, Tyr181 and Leu184 [38]. In the present study, our hits were observed to form bonds with the above-mentioned residues, characterized by hydrogen, hydrophobic and van der Waals interactions. Most notably, hydrogen bonds were perceived with residues Asp70, Gly108, Gly110 and Tyr120 of the RKIP binding pocket (Figure 6). Furthermore, interactions with residues Ala73, Pro74, Tyr81, His86, Val107, Gly108, Pro111, Pro112, His118, Leu180, Tyr181 and Leu184 were mostly driven by van der Waals, hydrophobic or carbon–hydrogen bonds (Figure S2). In order to identify the individual residues contributing considerably to the total binding free energy of each compound, the per-residue energy decomposition was estimated by MM/PBSA. The residues Trp84 and Tyr181 appeared to play an indispensable role in the affinity of all hits towards RKIP (Figure 7). These two residues were also identified as major contributing factors by Rudnitskaya et al. for RKIP/locostatin binding by MD simulation and quantum mechanics/molecular mechanics (QM/MM) [19]. The above overall analyses provide ample support for our hits as potential lead molecules to modulate RKIP. Individually, the identified hits portrayed the pharmacophoric features displayed by locostatin (Figure 3 and Figure S3).

As a final evaluation and on the basis of IUPAC names of thirteen drug-like compounds (Table S1), their source of origin was identified (Table 4). The hit compounds- HIT1, HIT2 and HIT5 were identified as alkaloids derived from a fungus *Stachybotrys* sp. [39], sponge *Psammaplysilla purpurea* [40] and annelid *Cirriformia tentaculata* [41], respectively. HIT3 was identified as a metabolite of a marine sediment and obtained from *Streptomyces* sp. [42], while HIT4 was isolated from the ascidian *Hypsistozoa fasmeriana* [42]. The chemical names and the source of additional molecules which demonstrated less binding free energy scores towards RKIP were also identified and reported in our study (Table 4). Overall, we believe that our hits can be utilized as potential alternatives to modulate the role of RKIP. Even though the experimental validation is required to validate our findings, auto-pharmacophore modeling from a single structure of a ligand can be helpful for designing potent molecules with similar efficacies. Additionally, our study represents a crucial platform for future drug optimization strategies from aquatic habitat.

## 4. Materials and Methods

### 4.1. Auto-Pharmacophore Model Generation

Locostatin is a well-known inhibitor of cell migration and cell–substratum adhesion, covalently binding RKIP and disrupting its association with Raf-1 kinase as well as GRK2 [19]. The α,β-unsaturated carbonyl functionality of locostatin renders it potently reactive towards RKIP and sterically hinders the binding of other ligands in the pocket [18]. Therefore, the chemical features shared by its 2-oxazolidinone core were exploited by employing the *Auto Pharmacophore Generation* module in Discovery Studio (DS) v.2018 (Accelrys Inc. San Diego, CA, USA). This module predominantly considers the hydrogen bond acceptor (HBA), hydrogen bond donor (HBD), hydrophobic (HyP), negative ionizable (NEG_IONIZABLE), positive ionizable (POS_IONIZABLE) and ring aromatic (RA) features to generate a selective pharmacophore model from a single ligand. Moreover, the module elects the pharmacophore with the highest selectivity depending on the prediction by Genetic Function Approximation (GFA) model.

### 4.2. Virtual Screening of Marine-Derived Natural Products

The auto-pharmacophore model generated from the above step was utilized as a 3D-query to retrieve the compounds, complementing the features of the model, from a Marine Natural Products (MNP) library. The MNP library comprising a total of 14,492 natural compounds was screened using the generated model by employing the *Ligand Pharmacophore Mapping module* in DS [48]. The resulting MNPs complementing the pharmacophore features were filtered by Lipinski’s Rule of Five (Ro5) [33,49] and Veber’s rules [34], followed by further filtering their absorption, distribution, metabolism, excretion and toxicity (ADMET) properties. Accordingly, the *Filter by Lipinski and Veber Rules* and *ADMET Descriptors* modules implanted in DS were employed for retrieving the drug-like MNPs. Subsequently, the obtained drug-like MNPs were escalated for the next process of molecular docking with the ligand-binding pocket of RKIP.

### 4.3. Molecular Docking of Drug-Like Molecules with RKIP Ligand-Binding Pocket

Molecular docking techniques are established in silico methods that are applied widely in drug discovery for identifying novel compounds of therapeutic interest and predicting their interactions within the catalytic sites of macromolecular target proteins [50]. The drug-like MNPs acquired from the above virtual screening were further subjected to molecular docking with the crystal structure of RKIP (PDB ID: 2QYQ) [36] in Genetic Optimisation for Ligand Docking (GOLD) v5.2.2 docking software (CCDC software ltd., Cambridge, UK) [51]. The drug-like MNPs were assessed on the basis of two default scoring functions, implanted in GOLD-Goldscore and Chemscore [52,53,54]. Prior to docking, the retrieved 3D crystallographic RKIP structure was prepared by employing the *Clean Protein* module in DS and further removing the water molecules as well as the bound o-phosphotyrosine (PTR). Consequently, both the RKIP protein structure and drug-like MNPs were minimized by employing the *Minimization* and *Minimize Ligands* modules in DS [55]. A total of 50 conformers per ligand were allowed to generate for the drug-like MNPs and locostatin, which was considered as reference (REF). Each compound was examined on the basis of its obtained conformation in the largest cluster, high Goldscore, low Chemscore and molecular interactions with the vital residues of the RKIP binding pocket. Only the drug-like MNPs demonstrating better scores than locostatin and similar interactions were retained from this process and escalated for molecular dynamics (MD) simulation studies.

### 4.4. Molecular Dynamics Simulation of Identified Marine-Derived Natural Products

MD simulation studies are widely used to provide the dynamical structural information on biomacromolecules as well as knowledge about protein–ligand interactions at the physiological level [56]. The docked complexes resulting from the above docking process were subjected to MD simulations with GROningen MAchine for Chemical Simulations (GROMACS) v2018 (University of Groningen, Netherlands; Royal Institute of Technology; Uppsala University, Uppsala, Sweden) [57]. The topologies for RKIP and the compounds were generated with CHARMm27 force field [58] and SwissParam (Swiss Institute of Bioinformatics) [59] fast force field generation tool, respectively. The solvation of all systems was performed via a dodecahedron water box and TIP3P (transferable intermolecular potential with 3 points) water model. Further neutralization of systems was carried out by supplementing them with Cl^-^ ions. Bad contacts in the systems were dodged by performing initial energy minimization followed by a two-step equilibration. The first step encompassed the NVT (constant number of particles, volume and temperature) equilibration at 300 K with a V-rescale thermostat for 500 ps. The second step involved NPT (constant number of particles, pressure and temperature) equilibration at 1 bar pressure with a Parrinello–Rahman barostat for 1000 ps [60]. Two algorithms, namely the LINear Constraint Solver (LINCS) [61] and SETTLE [62], were employed in order to monitor bond constraints and the geometry of water molecules. The long-range electrostatic interactions were computed by an *N*·log(*N*) method known as Particle mesh Ewald (PME) [63]. The systems after equilibration by both NVT and NPT were subjected to production simulation runs of 50 ns each. The results acquired after the production run were visualized in visual molecular dynamics (VMD, University of Illinois, Urbana, IL, USA) in order to interpret the stability of ligands in the RKIP pocket throughout the run [64]. Furthermore, the stability of all systems was also assessed by plotting their root mean square deviation (RMSD), root mean square fluctuation (RMSF) and potential energy plots for the entire 50 ns run [65].

### 4.5. Binding Free Energy Calculations of Identified Hits

The estimation of binding affinities of inhibitors with their macromolecular targets plays a quintessential role in drug discovery [66]. The binding free energy (BFE) estimation program, compatible with GROMACS, was utilized to predict the binding affinity of each ligand with RKIP. The molecular mechanics/Poisson–Boltzmann surface area (MM/PBSA) program is extensively utilized in drug discovery paradigms to compute the BFE of protein–ligand complexes and has been revealed to be a precise estimator in terms of correlation between experimental and theoretical values [67,68]. For computing this BFE, 25 snapshots of RKIP-ligand complexes were selected evenly from 40 to 50 ns of MD trajectories, and the resultant energy $\Delta G_{bind}$ was calculated on the basis of the following equation.

(1) $$\Delta G_{bind}=G_{complex}-\left(G_{protein}+G_{ligand}\right)$$

The resulting RKIP-compound complexes with better BFE scores than RKIP-locostatin were considered as hits from the present in silico investigation. 

## 5. Conclusions

An auto-pharmacophore model, exploiting the features of the most potent RKIP inhibitor, locostatin, revealed key pharmacophoric features imperative for binding RKIP. An orderly virtual screening process with the generated model as a 3D query, retrieved 2557 compounds from the Marine Natural Products (MNP) library and consequent filtration by Lipinski’s, Veber’s and ADMET was able to procure a total of 134 drug-like MNPs. The process of molecular docking of drug-like MNPs with the ligand-binding pocket of RKIP resulted in thirteen compounds with better docking scores than locostatin as well as noteworthy intermolecular interactions with vital residues of the pocket. From a total of thirteen compounds, only five demonstrated better binding free energy scores towards RKIP than that obtained for locostatin. Therefore, the five compounds were deemed as hit molecules from the current analysis. The per-residue energy contribution unveiled Trp84 as the most significant residue contributing to binding affinity towards RKIP. The biological origins of all thirteen compounds acquired from the present investigation was identified as either marine sponge, coral or fungus. Above all, we believe that our marine-derived hits provide scaffolds for future drug optimization studies against RKIP-related diseases. In conclusion, bioactive compounds from marine natural origin provide diverse scaffolds and represent a crucial platform for imminent drug discovery against various pathological complications.

## Figures and Tables

**Table 1:** Auto-pharmacophore model summary with its generated features.

Pharmacophore Model	Number of Features	Feature Set *
Pharmacophore hypothesis	4	2HBA, RA, HyP

* HBA: hydrogen bond acceptor; RA: ring aromatic; HyP: hydrophobic.

**Table 2:** The docking scores of reference (REF) compound, locostatin and Marine Natural Product (MNP) library compounds with RKIP ligand-binding pocket (PDB ID: 2QYQ).

Compound No.	MNP ID (CAS No *)	Goldscore	Chemscore
1	62541-09-7	67.72	−33.37
2	799246-91-6	64.48	−31.02
3	383191-01-3	60.85	−28.18
4	313951-44-9	59.03	−35.95
5	61897-90-3	58.61	−27.03
6	302924-16-9	58.39	−29.14
7	182806-09-3	58.37	−27.80
8	587875-53-4	57.76	−28.71
9	142677-12-1	57.18	−35.13
10	144385-02-4	57.07	−30.29
11	853885-48-0	56.93	−27.96
12	853885-46-8	56.04	−34.01
13	58115-31-4	55.01	−37.15
14	133812-16-5 (REF)	48.64	−26.48

* CAS: Chemical Abstracts Service.

**Table 3:** The entropic distribution of the total binding free energy (BFE) scores of reference (REF) compound, locostatin and identified hits from Marine Natural Products (MNP) library with RKIP (PDB ID: 2QYQ).

Hit No.	MNP ID (CAS No *)	Van Der Waals (kJ/mol)	Electrostatic (kJ/mol)	Polar Solvation (kJ/mol)	SASA Energy (kJ/mol)	BFE Scores ∆G_bind_ (kJ/mol)
HIT1	144385-02-4	−171.724 ± 13.242	−89.779 ± 8.171	143.371 ± 8.006	−17.151 ± 1.008	−135.283 ± 11.815
HIT2	799246-91-6	−159.666 ± 9.645	−66.487 ± 8.367	115.080 ± 11.961	−15.523 ± 0.756	−126.597 ± 8.883
HIT3	853885-46-8	−152.922 ± 10.576	−64.325 ± 7.461	117.843 ± 8.005	−15.684 ± 0.925	−115.088 ± 9.005
HIT4	383191-01-3	−144.389 ± 13.393	−68.213 ± 8.692	131.246 ± 6.782	−14.093 ± 0.641	−95.450 ± 10.777
HIT5	587875-53-4	−118.918 ± 10.507	−78.010 ± 7.488	114.660 ± 4.794	−12.315 ± 0.793	−94.582 ± 8.703
HIT6	133812-16-5 (REF)	−149.624 ± 7.721	−62.839 ± 5.691	134.966 ± 6.467	−13.413 ± 0.628	−90.909 ± 9.155

* CAS: Chemical Abstracts Service.

**Table 4:** Molecular structures and chemical source of identified marine-derived drug-like compounds.

HIT/CAS No *	Chemical Name (Source)	Reference	Molecular Structure
HIT1 (144385-02-4)	Stachybotrin B (*Stachybotrys* sp.)	[39]	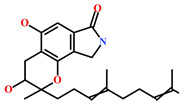
HIT2 (799246-91-6)	Purpurealidin G (*Psammaplysilla purpurea*)	[40]	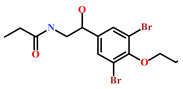
HIT3 (853885-46-8)	Glaciapyrrole A (*Streptomyces* sp.)	[42,43]	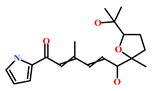
HIT4 (383191-01-3)	(*Hypsistozoa fasmeriana*)	[42]	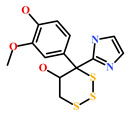
HIT5 (587875-53-4)	2-Hexylpyrrole sulfamate (*Cirriformia tentaculata*)	[41,42]	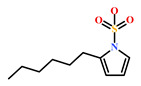
58115-31-4	Aurantiamide	[44]	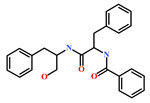
182806-09-3	Hemibastadinol 1 (*Ianthella basta*)	[45]	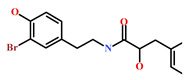
61897-90-3	Fumitremorgin H. (*Aspergillus fumigatus*)	[42]	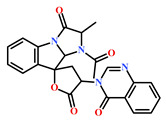
142677-12-1	(*Chondria* sp.)	[46]	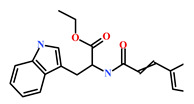
313951-44-9	Lorneamide A	[46,47]	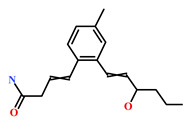
302924-16-9	Secobipinnatin J (*Pseudopterogorgia bipinnata*)	[42]	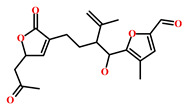
853885-48-0	Glaciapyrrole B (*Streptomyces* sp.)	[42,43]	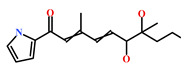
62541-09-7	Dehydrocoelenterazine (*Watasenia dehydropreluciferin*)	[42]	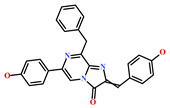

* CAS: Chemical Abstracts Service.

## Data Availability

Data are contained within the article.

## Supplementary Materials

The following are available online at https://www.mdpi.com/article/10.3390/md19100581/s1, Figure S1: Validation of GOLD docking parameters using co-crystallized ligand, PTR (orange) and its docked pose (pink). Figure S2: The two-dimensional (2D) intermolecular interactions of reference (REF) compound, locostatin and the identified hits with the key residues of RKIP. Figure S3: The mapping of identified Marine Natural Products (MNP) hits onto the generated pharmacophore model. All hits display the hydrogen bond acceptor (HBA), hydrophobic (HyP) and ring aromatic (RA) pharmacophoric features. Table S1: The binding free energy (BFE) scores of reference (REF) locostatin and drug-like Marine Natural Products (MNP) with RKIP (PDB ID: 2QYQ) along with their IUPAC names.
