# Supplementary material for: Investigation of Marine-Derived Natural Products as Raf Kinase Inhibitory Protein (RKIP)-Binding Ligands
Source: Mar Drugs. 2021 Oct 18;19(10):581. doi: 10.3390/md19100581 (PMC8539980; doi:10.3390/md19100581)
Supplement: Supplementary file 1 [file marinedrugs-19-00581-s001.zip › marinedrugs-1414445-supplementary.pdf]

marine drugs

MDPI

Article

Investigation of Marine-Derived Natural Products as Raf Kinase Inhibitory Protein (RKIP)-Binding Ligands

Shraddha Parate [1], Vikas Kumar [2], Jong Chan Hong [1,*] and Keun Woo Lee [2,*]

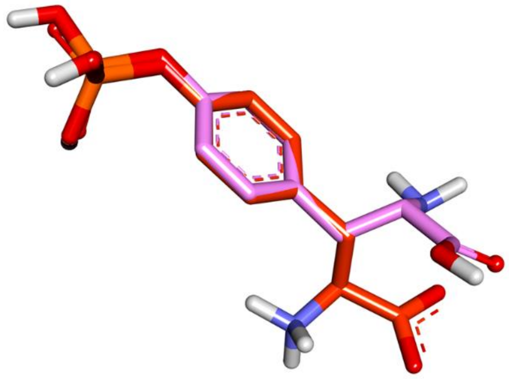

Figure S1. Validation of GOLD docking parameters using co-crystallized ligand, PTR (orange) and its docked pose (pink).

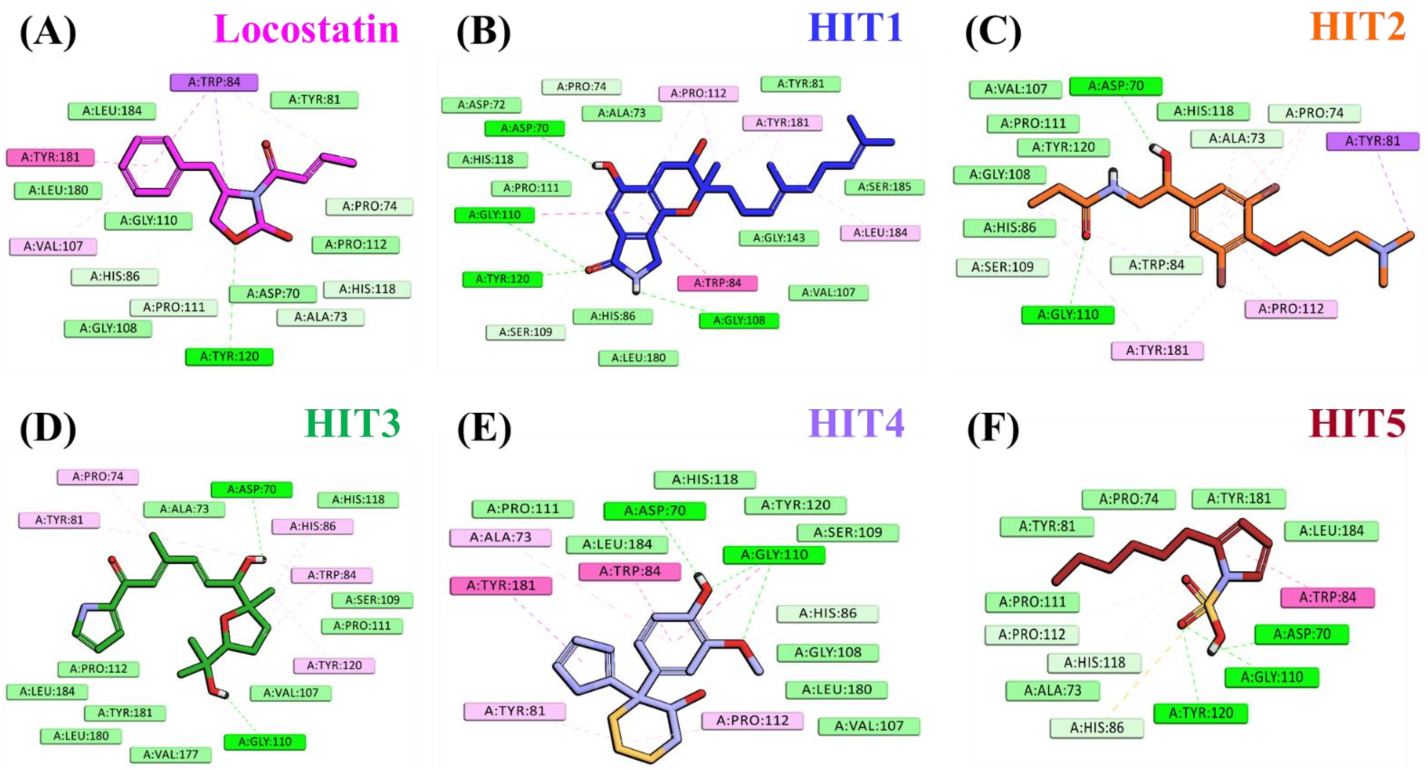

Figure S2. The two-dimensional (2D) intermolecular interactions of reference (REF) compound, locostatin and the identified hits with the key residues of RKIP.

Table S1. The binding free energy (BFE) scores of reference (REF) locostatin and drug-like Marine Natural Products (MNP) with RKIP (PDB ID: 2QYQ) along with their IUPAC names.

Compound No.	MNP ID (CAS No)	BFE scores ΔG_{bind} (kJ/mol)	IUPAC Name
1	62541-09-7	-34.910+/-39.452	(2Z)-8-Benzyl-2-(4-hydroxybenzylidene)-6-(4-hydroxyphenyl)imidazo[1,2-a]pyrazin-3(2H)-one
2	799246-91-6	-126.597+/-8.883	N-(2-{3,5-Dibromo-4-[3-(dimethylamino)propoxy]phenyl}-2-hydroxyethyl)propanamide
3	383191-01-3	-95.450+/-10.777	4-(4-Hydroxy-3-methoxyphenyl)-4-(1H-imidazol-2-yl)-1,2,3-trithian-5-ol
4	313951-44-9	-59.456+/-53.152	(3E)-4-{2-[(1E)-3-Hydroxy-1-hexen-1-yl]-4-methylphenyl}-3-butenamide
5	61897-90-3	-67.601+/-13.693	3-acetyl-2-methyl-3'-(4-oxoquinazolin-3-yl)spirol[2,3a-dihydroimidazol[1,2-a]indole-4,5'-tetrahydrofuran]-1,2'-dione
6	302924-16-9	-57.769+/-15.693	5-[(1S,2S)-1-Hydroxy-3-methyl-2-{2-[(5S)-2-oxo-5-(2-oxopropyl)-2,5-dihydro-3-furanyl]ethyl}-3-buten-1-yl]-4-methyl-2-furaldehyde
7	182806-09-3	-85.303+/-11.793	3-(3-Bromo-4-hydroxyphenyl)-N-[2-(3-bromo-4-hydroxyphenyl)ethyl]-2-hydroxypropanamide
8	587875-53-4	-94.582+/-8.703	2-Hexyl-1H-pyrrole-1-sulfonic acid
9	142677-12-1	-66.536+/-10.545	ethyl 3-(1H-indol-3-yl)2-[3-(1H-indol-3-yl)prop-2-enoylamino]propanoate
10	144385-02-4	-135.283+/-11.815	2-(4,8-dimethylnona-3,7-dienyl)-3,5-dihydro-2-methyl-3,4,8,9-tetrahydropyranol[2,3-e]isoindol-7-one
11	853885-48-0	-57.585+/-22.654	6,7-dihydroxy-3,7,11-trimethyl-1-(1H-pyrrol-2-yl)dodeca-2,4,10-trien-1-one
12	853885-46-8	-115.088+/-9.005	6-hydroxy-6-[5-(1-hydroxy-1-methyl-ethyl)-2-methyl-tetrahydrofuran-2-yl]-3-methyl-1-(1H-pyrrol-2-yl)hexa-2,4-dien-1-one
13	58115-31-4	-90.472+/-14.096	N-[1-benzyl-2[(1-benzyl-2-hydroxy-ethyl)amino]-2-oxo-ethyl]benzamide
14	133812-16-5 (REF)	-90.909+/-9.155	(4S)-4-Benzyl-3-[(2E)-2-butenoyl]-1,3-oxazolidin-2-one

marine drugs

MDPI

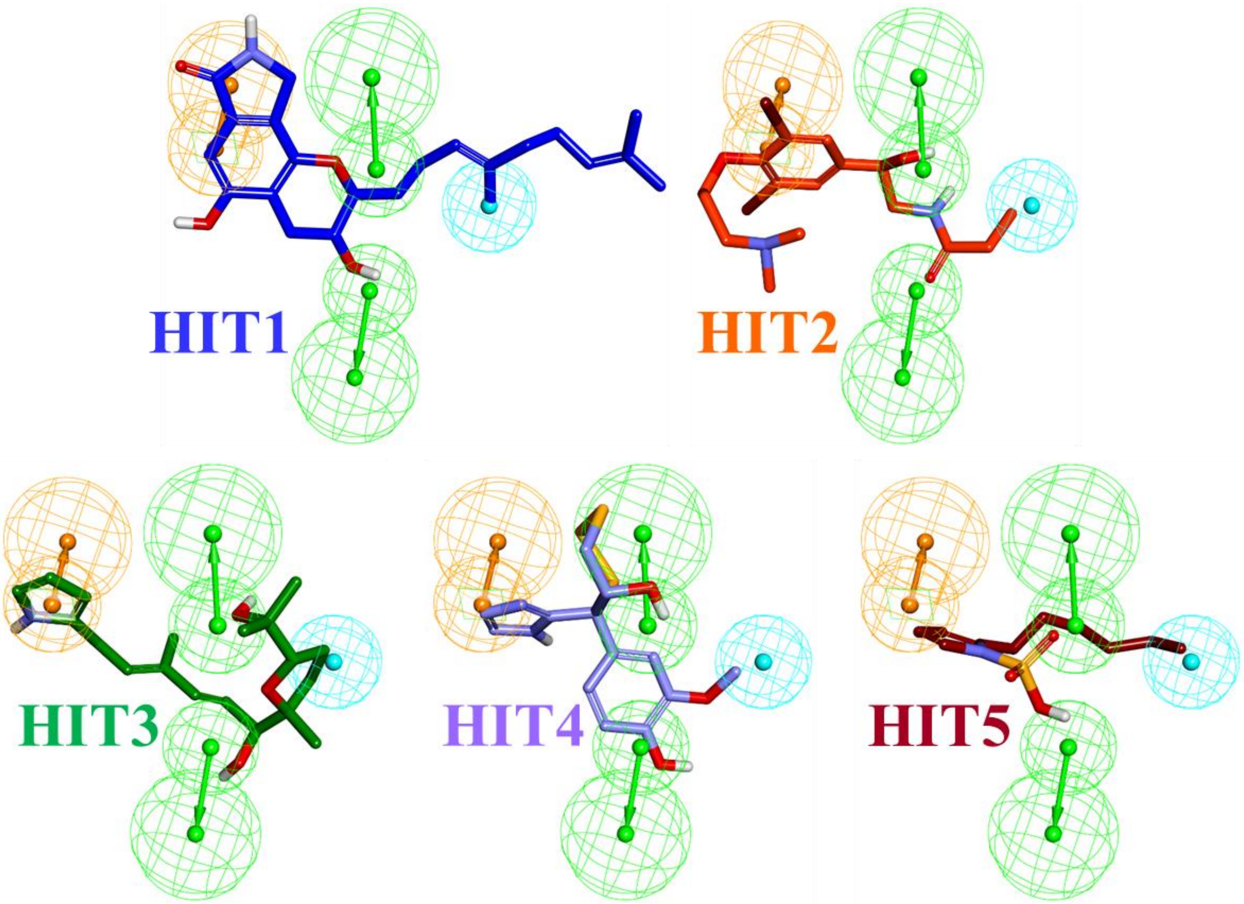

Figure S3. The mapping of identified Marine Natural Products (MNP) hits onto the generated pharmacophore model. All hits display the hydrogen bond acceptor (HBA), hydrophobic (HyP) and ring aromatic (RA) pharmacophoric features.
